# The use of the EQ-5D-Y health related quality of life outcome measure in children in the Western Cape, South Africa: psychometric properties, feasibility and usefulness - a longitudinal, analytical study

**DOI:** 10.1186/s12955-017-0590-3

**Published:** 2017-01-19

**Authors:** Des Scott, Gillian D. Ferguson, Jennifer Jelsma

**Affiliations:** Department of Health and Rehabilitation Sciences, Faculty of Health Sciences, University of Cape Town, Observatory 7925, Cape Town, South Africa

**Keywords:** HRQoL, Children, EQ-5D-Y, Psychometric properties, Health conditions

## Abstract

**Background:**

The EQ-5D-Y, an outcome measure of Health Related Quality of Life (HRQoL) in children, was developed by an international task team in 2010. The multinational feasibility, reliability and validity study which followed was undertaken with mainly healthy children. The aim of this study was to investigate the psychometric properties of the EQ-5D-Y when used to assess the HRQoL of children with different health states.

**Method:**

A sample of 224 children between eight and twelve years were grouped according to their health state. The groups included 52 acutely ill children, 67 children with either a chronic health condition or disability and 105 mostly healthy, mainstream school children as a comparator. They were assessed at baseline, at three months and at six months. An analysis of the psychometric properties was performed to assess the reliability, validity and responsiveness of the EQ-5D-Y in the different groups of children. Cohen’s kappa, the intraclass correlation coefficient, Pearson Chi-square, Kruskal-Wallis ANOVA and effect size of Wilcoxon Signed-rank test were used to determine the reliability, validity and responsiveness of the instrument.

**Results:**

The EQ-5D-Y dimensions were found to be reliable on test-retest (kappa varying from 0.365 to 0.653), except for the Usual Activities dimension (kappa 0.199). The Visual Analogue Scale (VAS) was also reliable (ICC = 0.77). Post-hoc analysis indicated that dimensions were able to discriminate between acutely ill and healthy children (all differences *p* < 0.001). The acutely ill children had the lowest ranked VAS (median 50, range 0–100), indicating worst HRQoL and was the only group significantly different from the other three groups (*p* < 0.001 in all cases). Convergent validity between all similar EQ-5D-Y and PedsQL, WeeFIM and Faces Pain Scale dimensions was only evident in the acutely ill children. As expected the largest treatment effect was also observed in these children (Wilcoxon Signed-rank test for VAS was 0.43). Six of the nine therapists who took part in the study, found the measure quick and easy to apply, used the information in the management of the child and would continue to use it in future.

**Conclusions:**

The EQ-5D-Y could be used with confidence as an outcome measure for acutely-ill children, but demonstrated poorer psychometric properties in children with no health condition or chronic conditions. It appears to be feasible and useful to include the EQ-5D-Y in routine assessments of children.

## Background

Self-reported HRQoL in children has become increasingly recognised as an important supplementary measure in assisting health professionals to assess the impact disease and intervention strategies have on the child’ life. This should be assessed from the child’s own perspective with self-reports [1–4]. The use of appropriate age related HRQoL measures when planning an intervention, ensures that the child’s priorities (which may be different from that of the health care professional [5]) are met. Studies comparing the child’s self-report with an adult proxy report, have found that there is often poor agreement between the two, lending weight to the importance of HRQoL being self-reported by the child to capture HRQoL from the child’s perspective [6–9]. These studies found that children do not prioritise disease-related symptoms and lack of physical ability, as negatively affecting their HRQoL, whereas the adult proxy does. Other studies have found that children prioritise the emotional impact a health condition has on their HRQoL, whereas adult proxies tend to under-report on this dimension [10–12]. Tracking changes in a child’s HRQoL over time enables clinicians to adapt their management appropriately as the maturing child’s health condition and needs change [13, 14].

The EQ-5D-Y was developed by an international task team in 2010 [15] to assess HRQoL in children. As this version was intended to be comparable with the adult version EQ-5D, the wording and layout of the EQ-5D was modified to ensure relevance and clarity for the cognitive developmental stage of children as young as eight years. The measure requires children to self-report on five dimensions of health, namely; Mobility (walking about), Looking After Myself (LAM), Usual Activities (UA), Pain or Discomfort (P/D) and being Worried, Sad or Unhappy (WSU). The child can choose between three levels of severity (1) no, (2) some or (3) a lot of problems, which result in a health profile. Additionally, a Visual Analogue Scale (VAS) allows the child to subjectively rate their overall HRQoL on a graduated scale, with 0 indicating worst health state imaginable and 100 indicating best health.

The EQ-5D-Y has been shown to be reliable and valid in healthy school children in the general population in countries such as Sweden [16], South African [17] and Spain [18] as well as in a multinational study [1], although different comparators were used in the studies e.g. paper, web based or adult proxy versions. A study in the United Kingdom indicated that the EQ-5D-Y was less reliable than the Child Health Utility 9D in younger children aged six to seven years and the content validity was less suitable in addressing issues important to children, but this may have been due to the age of the children [19].

There was no standardisation on the time interval between test and re-test when assessing reliability as it varied from seven to ten days in the multinational study [1] to morning/afternoon in the United Kingdom study [19]. The length of time between administrations could influence the reliability, but it is recommended to re-test before a change in HRQoL can occur [20].

The high ceiling effect in the dimensions noted in the studies on healthy children encouraged further testing in clinical contexts. There is, however, limited evidence of the use of the EQ-5D-Y in different disease groups. Some studies used a cross sectional study design to assess construct validity of the measure in children with one specific health condition and reported that the EQ-5D-Y was valid for children with Cystic Fibrosis [21], asthma [22] and diabetes mellitus [23]. Similar results were reported in an Italian study, which found good reliability and acceptable discriminant validity in children with and without Acute Lymphoblastic Leukaemia [26], and a Swedish study in children and adolescents with and without a variety of functional disabilities [16]. In contrast, other research, which compared the severity in one specific health condition or across different health conditions, found the EQ-5D-Y to have limited discriminant validity in children with varying levels of chronic health conditions [24] and with a range of orthopaedic conditions [25]. The VAS and the dimensions have been reported to discriminate in different participation groups. Discriminant validity on the VAS and not in dimensions scores was evident in a study with children of different levels of physical activity [27]. This is in contrast to a study with healthy children and children with long standing chronic disabilities, which reported discriminant validity on dimensions, with disabled children recognising their limitations on a dimensional level, but not reporting a lower VAS than their able bodied peers [8]. There are limited longitudinal studies using the EQ-5D-Y to assess HRQoL over a period of time but there is evidence that it is a responsive measure. Changes in the health needs of children with a variety of mental health problems were detected over three years [28]; as were changes in HRQol in children with and without Celiac Disease over one year [29]. The EQ-5D-Y was found to be as responsiveness as the KINDLE-R, but less responsive than the KIDSCREEN 10 in capturing changes in a group of children with a range of health conditions including obesity, diabetes and respiratory impairments over 20 months [30].

The feasibility and use of the EQ-5D-Y as part of routine patient data has not been fully explored. Routine use of the measure could improve health care, by guiding clinicians planning of interventions, ensuring that aspects important to the individual child are addressed. Clinicians might be unaware of the psychological effects the health condition has on the child and this is one way to improve responsiveness to this aspect of health [31–33]. Similarly shared decision making, with the use of the EQ-5D-Y could promote communication between the child and clinician and result in better adherence to an intervention regime. The effectiveness of the intervention could also be monitored through the use of the HRQoL measure.

The aim of this study was therefore to investigate the performance of the EQ-5D-Y in children with different health states.

The aims of this study were therefore to assess reliability, validity and responsiveness of EQ-5D-Y in children with arrange of health states, as well as feasibility for routine use in a South African setting.

## Methods

### Study design and setting

An observational, analytical cohort study with repeated measures was conducted. Four different research settings, each with children in different health states, different levels of severity and type of management, were used. All settings catered for children from a similar socio-economic background (low to middle income).

A mainstream school with typically developing, mostly healthy children living in the surrounding areas was chosen as a comparator (MS group). Two facilities for chronically ill children were chosen, as the level of physical disability and management of the conditions was different at each facility. A Special School catering for children diagnosed with a congenital, physical disability limiting mobility, such as spina bifida, muscle diseases or cerebral palsy and relying on either a wheelchair or assistive device for mobility, was used. The management of their health condition was aimed at maintaining their limited functional ability through nursing and rehabilitative services, which were provided at the school (SS group). The second chronic care facility, cared for children with an acquired chronic condition, such as diabetes mellitus, human immunodeficiency virus (HIV), neurological disorders and heart, renal and respiratory impairments. The children were all independently ambulant, but were admitted to the facility as their families could no longer provide the level of nursing care needed. The improved care they received was aimed at improving their health (CI group). The fourth setting was an acute care paediatric hospital managing acutely ill children in intensive care or in medical, surgical, trauma or oncology wards. Most of these children were healthy before being admitted to hospital for acute appendicitis, septic arthritis, leukaemia or a bone fracture to name a few conditions. They were usually hospitalised for no longer than seven days, as they were discharged once the acute condition had improved (AI group).

### Participants

There were two main groups of participants, children with varying levels of health and their treating therapists.

The sample size was recalculated based on an anticipated moderate effect size between groups, as one of the aims was to determine whether the EQ-5D-Y could detect a change in health over time.

A one-way ANOVA using G*Power 3.1.9.2 calculator (http://www.ats.ucla.edu/stat/gpower) with an effect size of .3 (moderate difference between groups), an alpha error probability of .05 and power of .8, was used. The total sample required was 190, which was exceeded in recruitment.

All children at each facility between the ages of eight and 12 years, were identified and recruited during an onsite visit. This age range was chosen in order to assess the outcome measures’ performance in children specifically, as it was felt that adolescents (13–18 years) would have less problems in completing the questionnaires. Only children who returned legible, signed consent and assent forms were included in the study. Children who were in the terminal stages of illness or were critically ill, with unstable and/or abnormally high or low vital statistics were excluded as it was possible that they and their parents might find participating in the research study too distressing. Any child who was unable or unwilling to respond was excluded.

There were 105 mostly healthy children from a mainstream school recruited. Five of these children were diagnosed with relatively minor health issues such as asthma, eczema and headaches. They were not expected to have HRQoL problems and their health state was expected to remain stable throughout the study period. The 119 children with a health condition were divided into three groups as each group demonstrated a different level of disease severity and expected outcome. The SS group of 35 children with a stable, chronic disability all experienced problems with mobility which was not expected to change over time. The 32 children in the CI group with an acquired chronic health condition faced greater emotional challenges adjusting to their acquired health status, compared to the children born with a disability (SS group). Their health state was expected to improve moderately over time as a result of the improved care they received. The 52 acutely ill, hospitalised children, AI group, experienced problems in all HRQol dimensions, but this was expected to improve the most over time, with management of their acute condition.

A convenience sample of nine therapists was interviewed to determine the feasibility and use of the EQ-5D-Y, as a routine outcome measure. These were the therapists treating the children with a health condition at the various facilities.

### Measurements

A self-designed contextual information sheet, was used to record the demographics, general health status and management of the health condition of each child.

#### EQ-5D-Y

The EQ-5D-Y self-report was the primary outcome measure. The five dimensions, each with one item, do not share the same underlying construct and the intervals between the three levels of problems, are not necessarily equal, so the dimensions were analysed individually. Until recently, there was no single index score for the EQ-5D-Y summarising the level of problems reported on each dimension. Craig et al. [34] developed a summary EQ-5D-Y on a QALY (Quality Adjusted Life Years) scale which was used to determine a Composite Score, for comparison with other HRQol measures which do provide for a total dimension score. The QALY is a single index obtained by combining the length of time spent in a particular health state and the HRQoL weighting or utility score given to that health state. The QALY values produced by Craig et al. were based on adult preferences for child health states. Adults were asked to choose between losses in HRQoL and losses in life span, in children with a health condition. Paired comparisons of different health states were used to identify the point at which participants were indifferent between the choices. At this point, weights or the value attributed to the loss in HRQoL, assuming it lasts for one year, informed the QALY value. However, this has not yet been endorsed by the EuroQoL Research Foundation [http://www.euroqol.org/]. In addition the VAS allows for a subjective overall rating of health status on a graduated scale. The higher the reported VAS, the better the HRQoL [1].

#### The paediatric quality of life inventory (PedsQL4.0)

The generic PedsQL consists of self-reporting on 23 items, in four dimensions; About My Health and Activities (Physical Functioning e.g. “It is hard for me walk more than one block”) – a total of eight items, About My Feelings (Emotional Functioning e.g. “I feel afraid or scared”) – five items, How I Get Along With Others (Social Functioning e.g. “Other kids tease me”) – five items and About School (School Functioning e.g. “I miss school because of not feeling well) – five items. The Likert type scoring ranges from 0 to 4 with 0 being “never a problem”, and 4 “almost always a problem”. Each item is scored and the scores were added together to produce a dimension sub-score. Therefore the higher the PedsQL score, the greater the problem in that dimension and the lower the HRQoL [35].

#### Faces Pain Scale (FPS)

The FPS, with a series of facial expressions depicting pain intensity, was also completed by the children. A horizontal line of six faces, showing progressively worsening pain expressions, are used to self-rate pain. The first face shows “no pain” = 0. The next four faces show increasing pain, rated 2, 4, 6, 8 respectively; and the last face shows “very much pain” = 10. The child marks the face indicating his/her pain level. A rating of 10 would indicate severe pain [36].

#### WeeFIM

The observational/interview WeeFIM instrument was completed by the researcher and not self-reported on as all the other outcome measures. The WeeFIM gives an indication of the child’s functional independence, in three dimensions, namely self-care (e.g. eating, grooming etc.) with a total of eight items, mobility (e.g. transfers from bed to chair or wheelchair) with five items, communication (e.g. comprehension) with two items and cognition (e.g. memory) with three items. The 18 items are each rated on an ordinal scale from 1 to 7, ranging from complete dependence (rated as “1”) to complete independence (rated as “7”). The scores are summed and therefore the higher the dimension sub-score, the more independently the person is able to perform functions in that dimension [37]. The WeeFim was only completed for children with health conditions, and for the few MS children who indicated a problem with “Mobility” on the EQ-5D-Y.

#### Feasibility and utility questionnaire

The clinical therapists involved in the study completed a questionnaire designed by the researcher. The time taken to complete the measure was recorded and compared to the recommended time of five minutes. The therapists were asked to comment on the use of the EQ-5D-Y as a supplement to routine patient assessments. Questions included whether additional psychological information of which they were possible unaware, was obtained; whether there was a relationship between children’s responses and observed clinical signs; whether they used the measure to assist in planning of interventions and whether they would continue to use the measure in the future.

### Procedure

Ethical approval from the Human Research Ethics Committee of the University of Cape Town (HREC REF 354/2013) and permission from the various institutions at which the study took place were obtained. Letters were sent home to parents with children at the various schools. The letters explained the purpose of the study and included informed consent forms for parents to sign and assent forms for children. In the acutely ill, hospitalised children consent and assent forms were signed during face to face interviews with children and their parents at the bedside. The participating therapists also signed informed consent forms, during face to face interviews at each facility.

A pilot study, using the test-retest method on consecutive days, was conducted on a sub-sample of 38 children from the four different health groups to establish reliability of the EQ-5D-Y, in this population group.

Subsequently demographic information was collected on all 224 children. All outcome measures were administered on the same day, to small groups of eight children at a time, in a quiet room. In the hospitalised children this was performed individually at the bedside. Although all the children could understand English, some children chose to complete the outcome measures in their home language, Afrikaans. No child chose the isiXhosa versions, even though they were available. Afrikaans and isiXhosa are two of the 11 official languages of South Africa. The EQ-5D-Y was completed first by each child, followed by the PedsQL and FPS, with a short break between measures. The researcher then completed the WeeFIM for all SS, CI and AI children, but only for the MS children who indicated a problem with “Mobility” on the EQ-5D-Y. This was done to assess whether or not they actually did have a functional problem. The MS children were healthy children, with no functional problems and were therefore expected to show a ceiling effect in WeeFIM scores.

Baseline measures for the AI children were taken two days post hospital admission, allowing the child time to settle in, and repeated just prior to discharge, approximately five days later. A third assessment was taken at either two weeks or one month later, if the child was still hospitalised. Only the longer stay, AI children were therefore assessed three times. The other three groups were assessed at baseline, three months and six months after baseline. All repeat assessments followed the same procedure.

At the end of the study, the therapists completed the feasibility and utility questionnaire.

### Statistical analysis

Descriptive statistics were used to describe the sample. A One Way ANOVA was used to determine whether there were significant differences in mean ages of the children. A Chi-squared test (Chi^2^) was used to determine whether gender was significantly associated with health group.

Cohen’s unweighted, kappa coefficient of agreement between the children’s responses for EQ-5D-Y dimensions, tested 24 h apart used to assess reliability. Kappa values were interpreted according to Landis and Koch’s guidelines [38] with kappa <0.2 indicating poor agreement, 0.21–0.40 indicating fair agreement, 0.41–0.60 moderate agreement, 0.61–0.80 substantial agreement, and kappa >0.81 indicating almost perfect agreement.

The VAS scores were not normally distributed (KS < 0.01 throughout) and non-parametric analysis was used to compare the groups. A two-way mixed effects model, type A Intraclass Correlation Coefficient (ICC) for absolute agreement (95% Confidence Interval) for VAS was used to assess reliability of VAS scores. An ICC >0.7 was generally considered as acceptable for test–retest reliability [1].

Discriminant validity of the EQ-5D-Ywas examined by comparing the HRQoL profiles of the different groups of children, known to be different (healthy, chronically disabled, acquired chronic conditions and acutely ill children). The level of problem reported in each EQ-5D-Y dimension, per group, was compared using the Fisher’s exact test, rather than Chi^2^ test as some cells were sparsely populated. Post hoc analysis, using Kruskal-Wallis H test, indicated which groups were significantly different from each other.

Concurrent validity was assessed by comparing the EQ-5D-Y dimension profiles summarised using the Composite Score, with the self-perceived global perception of health, VAS. The Composite Score was calculated using the QALY weightings suggested by Craig et al. [34]. Spearman’s Rho was used to determine correlations between Composite Score and VAS. In line with the guidelines provided by Cohen [39], correlations from 0.1 to 0.29 were considered low, 0.3 to 0.49 moderate and correlations of 0.5 or above as high.

Convergent validity of the EQ-5D-Y dimensions was examined by correlating the dimension scores for the different groups with scores on similar dimensions of the PedsQL, WeeFIM and the FPS. Kruskal-Wallis H value was determined. When there were five or fewer scores for a particular problem level (1, 2 or 3) on the independent EQ-5D-Y variable, this level was excluded and the Mann–Whitney *U* Test was used to compare the remaining two levels. Spearman’s Rho was used to determine correlations between EQ-5D-Y Composite Score and VAS.

Responsiveness of the EQ-5D-Y to depict a change in health in repeat measures taken three months post baseline, was described by examining the effect size (r) of Wilcoxon Signed-rank test (Z). It was calculated by $\mathrm{r}=\frac{z}{\sqrt{N}}$ ^1^ where N is the total number of responses at baseline and at three months, not the number of participants. Effect size was interpreted as 0.1 being considered small, 0.3 medium and 0.5 large.^1^

Feasibility was assessed by the time taken to complete the EQ-5D-Y. The usefulness of the measure was assessed by analysing the frequency of positive responses in the questionnaire completed by the participating therapists.

## Results

**Fig. 1:**
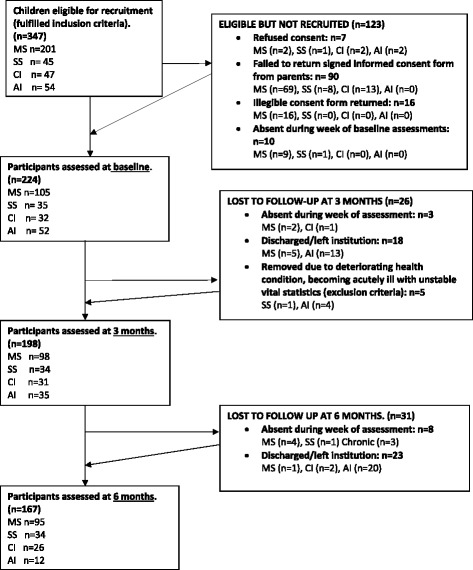
Flow diagram indicating the number of participants at each stage of the study

Figure 1 there were no missing responses on the EQ-5D-Y or PedsQL, as the researcher asked the child whether the missing response was due to the child not wanting to provide a score for that item or if the child had forgotten to provide a score for the item. The few children with missing responses did so inadvertently and were willing to provide the missing score, without coercion.

The mean age was 10.5 years (SD = 1.45) and there was no significant difference in age between the four groups of children F = (3, 220) = 1.03, *p* = .379. Gender distribution was also not significantly associated with any group (Chi^2^ = 1.43; *p* = .698).

Table 1 indicates the health status of the different groups. As was expected, the majority of the MS children did not have a health condition, although eight reported minor ailments (asthma, eczema and headaches). All the SS children were diagnosed with a disability which limited their functional independence e.g. cerebral palsy (*n* = 12), spina bifida (*n* = 10). The CI children with no mobility limitations were admitted for management of a chronic health condition e.g. HIV (Human immunodeficiency virus) (*n* = 8), diabetes mellitus (*n* = 8). The most common conditions seen in the AI group were appendicitis (*n* = 7), joint injuries (*n* = 7) and neoplasms (*n* = 6).

**Table 1:** Health status of participants in each group

Group	Chronic Health status	Acute Health status	No health condition	Totals (*n*)
MS	8 (7.6%)	0	97 (92.4%)	105
SS	35 (100%)	0	0	35
CI	32 (100%)	0	0	32
AI	^a^3 (5.8%)	49 (94.2%)	0	52
All Groups	78 (34.8%)	49 (21.9%)	97 (43.3%)	224

*MS* Healthy mainstream school children, *SS* Children with a chronic physical disability, *CI* Chronically ill children, *AI* Acutely ill children

*n* = 224

^a^These three children were treated for an acute health problem, unrelated to their chronic condition

### Reliability

#### Test-retest reliability of EQ-5D-Y dimension scores

This was performed in a pilot study, on a small sample of convenience of children from each of the four health groups. Nine MS children, five SS and nine CI children and 15 AI children, completed questionnaires, 24 h apart. Table 2 shows Mobility, LAM and WSU dimensions fell within the moderate level of agreement, while P/D indicated fair agreement, across all four groups. Only UA dimension fell within the slightly agreed category (kappa = 0.199, *p* < .127).

**Table 2:** Agreement between first and second EQ-5D-Y dimension scores

	Kappa	Strength of agreement	*p*
Mobility	.546	moderate	***p < .001***
LAM*	.653	good	***p < .001***
UA*	.199	poor	*p* < .127
P/D*	.365	fair	*p* < .08
WSU*	.551	moderate	***p < .001***

*n* = 38

*LAM (Looking After Myself), *UA (Usual Activities), *P/D (Pain/Discomfort), *WSU (Worried, Sad or Unhappy)

Significant *p* values are bolded

#### Test-retest reliability of VAS scores

The intraclass correlation coefficient (ICC) for all groups for VAS was found to be .765 (95% Confidence intervals (CIs) = .594-.870), which indicated strong agreement between the two sets of VAS scores.

### Discriminant validity

Discriminant validity was examined by comparing the responses on the different dimensions across the groups of children with different health states.

#### EQ-5D-Y dimensions

##### Mobility dimension

**Fig. 2:**
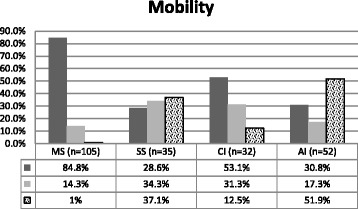
Percentage of Mobility dimension problems for each group. *n* = 224 *p* < .001 i.e., *p* = 1.3E-17. Fisher’s exact test, ***p < .001***. Kruskal-Wallis test: H (3, *N* = 224) =71.058 ***p < .001***

Figure 2 discriminant validity was evident between the AI group with significantly more reported problems with Mobility and the other three groups. The SS group reported significantly more Mobility problems than the MS group, demonstrating discriminant validity between these two groups in this dimension.

##### Looking After Myself (LAM) Dimension

**Fig. 3:**
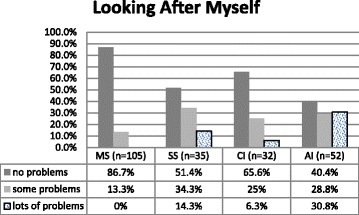
Percentage of LAM dimension problems for each group. *n* = 224. Fisher’s exact test, ***p < .001***. Kruskal-Wallis test: H (3, *N* = 224) =45.349 ***p < .001***

Figure 3 the SS and AI groups reported significantly more problems with Looking After Myself, than the MS group with few problems, but the SS and AI groups were not significantly different from each other.

##### Usual Activities (UA) dimension

**Fig. 4:**
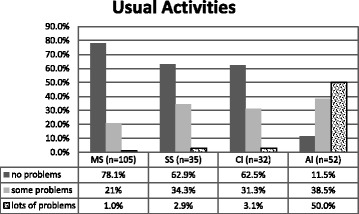
Percentage of UA dimension for each group. *n* = 224. Fisher’s exact test, ***p < .001*** **.** Kruskal-Wallis test: H (3, *N* = 224) =85.311 ***p < .001***

Figure 4 the AI group reported significantly more problems in the Usual Activities dimension compared to the other groups.

##### Pain/Discomfort (P/D) dimension

**Fig. 5:**
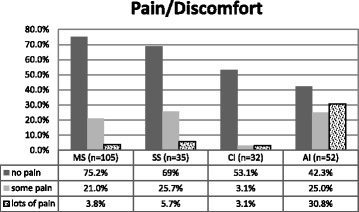
Percentage of P/D dimension problems for each group. *n* = 224. Fisher’s exact test, ***p < .001***. Kruskal-Wallis test: H (3, *N* = 224) =21.030 ***p < .001***

Figure 5 the only groups significantly different from each other were the AI group with significantly more pain than the MS group.

##### Worried, Sad or Unhappy (WSU) dimension

**Fig. 6:**
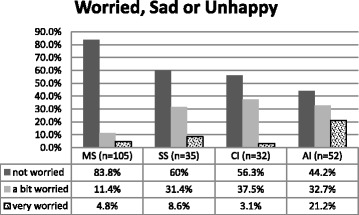
Percentage of WSU dimension problems for each group. *n* = 224. Fisher’s exact test, ***p < .001*** **.** Kruskal-Wallis test: H (3, *N* = 224) =25.895 ***p < .001***

Figure 6 again the only groups significantly different from each other were the AI with more Worried, Sad or Unhappy dimension problems than the MS group.

Overall the AI children reported the most problems on level 3 for all dimensions. The MS children reported the least problems in all dimensions apart from WSU dimension, in which 4.8% indicated problems on level 3 (lots of problems), compared to only 3.1% of CI children.

#### EQ-5D-Y composite scores

The Composite Score, summarising dimension profiles and calculated using the QALY values suggested by Craig et al. [34], was compared across groups. Note that the higher scores indicate more problems on the dimensions and worse HRQoL.

**Fig. 7:**
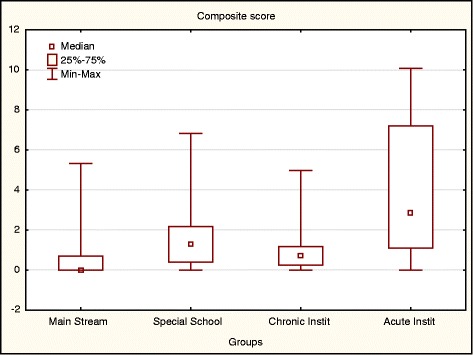
Median Composite Score per group. *n* = 224. Kruskal-Wallis test: H (3, *N* = 224) =72.86 ***p < .001***

Figure 7 indicated that the MS children with a median Composite Score of .15, experienced significantly fewer problems on dimensions compared to the AI children with a Composite Score of 2.8 with significantly more problems and worse HRQoL. There was no discriminant validity between the two groups of children with chronic health conditions, the SS and the CC groups (Composite Scores of 1.4 and .75 respectively).

#### EQ-5D-Y VAS scores

**Fig. 8:**
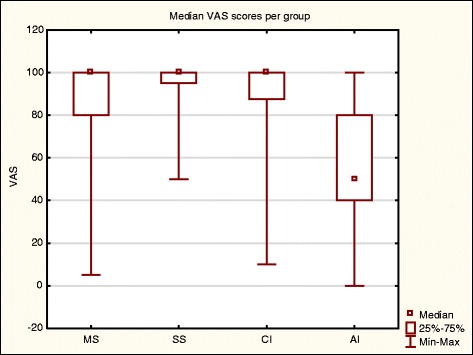
Median VAS per group. *n* = 224. Kruskal Wallis (H (3, *N* = 224) =62.81 ***p*** **< .001**)

The AI group VAS (median of 50) was ranked significantly lower than and the other three groups (all with a median of 100) and not ranked significantly different from each other (Fig. 8).

### Concurrent validity

The dimension profiles as summarised by the Composite Score were compared with the self-perceived global perception of health, VAS, to assess concurrent validity.

One outlier was removed from the CI for the scatterplot only. Removing the outlier from the scatterplot did not change the results, as can be seen in Spearman’s correlation below. The outlier was included in all other analyses.

**Fig. 9:**
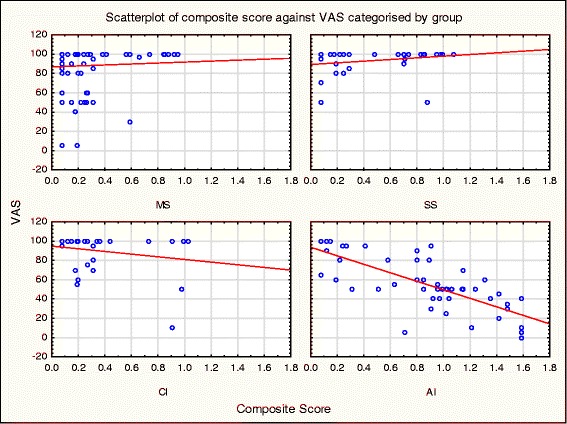
Scatterplot of VAS versus Composite Score

There was no correlation between the VAS and Composite Score in any group apart from the AI children (Fig. 9 and Table 3).

**Table 3:** Spearman Rank Order Correlations between VAS and Composite Scores across groups

	*n*	Spearman Rho	*P* value
MS	105	-.047	.638
SS	35	.304	.075
CI	32	-.202	.268
AI	52	−0.786	***p < .001***

Significant *p* values are bolded

### Convergent validity

Convergent validity of the EQ-5D-Y was examined by correlating the dimension scores for the different groups with their scores on similar dimensions of the PedsQL, WeeFIM and the FPS. Correlations between EQ-5D-Y Composite Score, VAS, PedsQL and WeeFIM total score were also examined (Table 4). It should be noted that the MS group were excluded from the analyses using the WeeFIM, as these children did not demonstrate a functional limitation.

**Table 4:** Summary of significant correlations between the different instruments per group

Group	Instrument	EQ-5D-Y dimensions					EQ-5D-Y VAS	EQ-5D-Y Composite Score
		Mobility	LAM	UA	P/D	WSU		
MS	PedsQL “Feelings”					***p = .02*** −2.318^b^		
	PedsQL total			***p = .007*** −2.682^b^				***p = .041*** Rho .199
SS	PedsQL “Health and Activity”	***p < .001*** 16.7^a^						
	WeeFIM Mobility	***p < .001*** 22.12^a^						
	WeeFIM self-care		***p < .001*** 14.19^a^					
	PedsQL total							***p = .009*** Rho .441
CI	WeeFIM Mobility	***p = .01*** 9.19^a^						
	WeeFIM Self-care		***p = .013*** 8.69^a^					
	PedsQL total			***p = .002*** −3.043^b^			***p = .002*** Rho-.523	
	WeeFIM total							***p = .024*** Rho -.398
AI	PedsQL “Health and Activity”	***p < .001*** 15.81^a^						
	PedsQL “I hurt”				***p < .001*** 26.78^a^			
	PedsQL “Feelings”					***p = .007*** 12.14^a^		
	WeeFIM Mobility	***p < .001*** 21.75^a^						
	WeeFIM Self-care		***p < .001*** 15.57^a^					
	FPS				***p < .001*** 29.76^a^			
	PedsQL total			***p = .006*** 10.335^a^			***p < .001*** Rho-.564	***p < .001*** Rho .635
	WeeFIM total						***p < .001*** Rho .525	***p < .001*** Rho-.659

^a^Kruskal-Wallis H value

^b^Mann–Whitney U z value

Significant *p* values are bolded

It would seem that all similar EQ-5D-Y and PedsQL dimensions were correlated in the AI group only. All similar EQ-5D-Y and WeeFIM dimensions were correlated for SS, CI and AI children. The EQ-5D-Y Pain/Discomfort dimension the Faces Pain Scale were correlated in the AI group only. There were significant correlations between total scores of all outcome measures for the AI group, only.

### Responsiveness of EQ-5D-Y over time

The ability of the EQ-5D-Y to depict a change in HRQoL between baseline and three months was examined to determine the responsiveness of the measure. Responsiveness was described by examining the effect size effect size (r) of Wilcoxon Signed-rank test (Z), calculated by $\mathrm{r}=\frac{z}{\sqrt{N}}$ where N is the total number of responses at baseline and at three months (Table 5).

**Table 5:** The effect size of the EQ-5D-Y Composite Score and Vas Score between baseline and 2^nd^ assessment, across groups

	MS	SS	CI	AI
EQ-5D-Y Composite Score	.02 Small effect size	.15 Small effect size	**.38** **Medium effect size**	**.41** **Medium effect size**
EQ-5D-Y VAS	.15 Small effect size	.08 Small effect size	**.36** **Medium effect size**	**.43** **Medium effect size**

Significant *p* values are bolded

The Composite Scores and VAS scores over time were examined for responsiveness.

The Composite Scores and VAS both indicated medium effective size and responsiveness for both CI and AI groups, the two groups at which change was expected, due to management of the children’s health conditions.

### Feasibility and usefulness of EQ-5D-Y outcome measure

All the children completed the EQ-5D-Y within the recommended time of five minutes.

Nine clinical therapists assisted in administering the EQ-5D-Y to some children. Six of the therapists found the measure very easy to use. The reported reason for three therapists finding it only moderately easy to use was time restraints. Two therapists reported that eight and nine year- old AI children had some difficulties understanding the UA dimension. A relationship between responses and objective clinical signs was mostly noticed in the EQ-5D-Y Mobility dimension and ability to walk followed by P/D and WSU. All therapists found the measure useful in planning the management of the child, especially the information on P/D and WSU. Six of the therapist agreed that they would continue to use the instrument to assist the planning and monitoring of an intervention.

## Discussion

As the measure has been found to be reliable in other studies on children with and without health conditions [1, 16, 18, 26] including in South Africa [17], a pilot study with 38 children was used to confirm reliability in the four groups. Fair to good test-retest agreement in EQ-5D-Y dimensions except for the UA dimension, was demonstrated in the small sub group of children chosen from each of the four facilities. Of concern was the lack of consistency in the UA dimension. It would seem that the child might have been relating to a different, specific usual activity each time, as several examples of usual activities are included in the questionnaire to explain the construct (going to school, hobbies, sports, playing, doing things with family or friends and…). Further qualitative research would be required to determine the childrens understanding of this dimension, backed up by a reliability study on a larger sample than in this paper.

The agreement between VAS scores was rated as good, in the small sample. The time interval of 48 h between test and retest was deemed appropriate as the health status was likely to change in the AI children, over a longer period, but was long enough to ensure that the children would not remember their initial score, as recommended by Devon et al. [40].

The EQ-5D-Y performed the best in acutely-ill children and the measure was able to discriminate between them and all the other groups of children, whether assessing dimensions, Composite Scores or the VAS. It seems that children with an acute health condition were able to respond most appropriately to the EQ-5D-Y, reporting accurately on the impact of their health condition in dimensions and VAS. They reported the most problems on all dimensions and the Composite score, summarising dimension scores, correlated with lowered VAS scores, despite the composite score being based on adults valuing health losses of a child and the VAS being the child’s perception of their own health. An Italian study also found that the EQ-5D-Y could differentiate between children from the general population and children diagnosed with Acute Lymphoblastic Leukaemia, who reported more problems on four of the five dimensions (with the exception being Mobility) and lower VAS [26].

It was expected that the mostly healthy MS children would report relatively few problems with a high ceiling effect as was found in other studies with children from the general population [1, 19, 21, 26, 41]. While over 75% of these children did report no problems on any dimension and a median VAS of 100, there were some unexpected results in the Mobility and Pain and Discomfort dimensions. Some of these children (14%) reported problems with Mobility on the EQ-5D-Y, but this was not reflected when they were assessed by the researcher on the functional independence outcome measure, the WeeFIM. This may reflect an interpretation and contextual issue with the EQ-5D-Y in these children. It would seem that they did not always relate problems with “Mobility (Walking About)” to a health state, but rather to environmental barriers, such as a lack of safety in the high crime areas in which they lived. Additionally, unexpectedly high numbers of MS children (21%) reported some problems in the Pain or Discomfort dimension, but this was not evident when they reported pain levels on the Faces Pain Scale. It could be that the children were reporting on relatively minor and transient pain on the EQ-5D-Y, as only two of the five children with asthma, eczema or headaches reported pain on the EQ-5D-Y. There was also a lack of correlation between the Composite Score, summarising dimension scores and VAS in the MS children which could be attributed to the Composite Score being based on adults valuing health losses of a child, but this did not affect the results found in the acutely ill children. It is possible that children who have not experienced a serious health condition may not be able to differentiate between *health* related quality of life and *general* quality of life. This could affect the validity of the EQ-5D-Y in healthy children. These findings suggest that the EQ-5D-Y should be used with caution in children with transient, minor health conditions or no health condition and that the measure may be limited in detecting moderate impairments or discriminating between groups of healthy children.

It is acknowledged that the sample size of the children with chronic disabilities and chronic acquired conditions (35 and 32 respectively) was small, which is a limitation of the study. From the results it would appear that this small sample of children were able to recognise and report their problems appropriately on the five EQ-D-Y dimensions. However, similar to another South African study [8], the VAS of these children was equivalent to the MS children, with a median of 100, which does not reflect the level of problem reported on dimensions. The reasons for this disparity need further investigation to understand how children with chronic conditions conceptualise health on the VAS. It could be that hedonic adaptation [42] takes place whereby the children with chronic conditions recalibrate their perception of good health [43]. This was also reported on in a German study assessing the validity of the EQ-5D-Y in children with cystic fibrosis. These authors suggested that the children learned to cope with the limitations imposed on their HRQoL by the disease and did not perceive this as a negative effect [21]. Alternative explanations could be that other constructs underpin the children’s views of their health, such as their satisfaction with participation in their school environment and strong social supports within this context, despite problems on a dimensional level. Another limitation in using the EQ-5D-Y in children with a chronic condition is that discriminant validity was not evident between the two groups with chronic conditions, despite significant differences in their Mobility, however the sample size was small. A Swedish study assessing the use of the EQ-5D-Y in children with chronic functional disabilities and healthy children, found that the children with chronic disabilities also reported significantly more problems on dimensions and lower VAS, but discriminate validity between chronic conditions was not analysed [16].

Concurrent validity between the EQ-5D-Y Composite Score and VAS and the other outcome measures (PedsQL, WeeFIM and FPS) total scores, was demonstrated in the AI children only. This further supports the validity when using the EQ-5D-Y in acutely ill children. It should be noted that the EQ-5D-Y Composite Score is not ideal as it is derived from an adult valuing losses in a child’s health QALY value and has not yet been formally endorsed by the EuroQoL group. A few paediatric studies have used the Composite Score, but these studies have been conducted for economic evaluations and resource allocation purposes [44–47].

Convergent validity was evident between all similar EQ-5D-Y and PedsQL dimensions in the AI group only. This pattern of association was not evident in the other groups of children and could possibly be attributed to fewer variations in EQ-5D-Y scores, in these groups of children compared to AI children. However, for all groups tested (SS, CI and AI) the convergent validity against the WeeFIM, which the researcher completed, was high (rho = −0.60) indicating that the children were able to reliably describe their functional problems on the EQ-5D-Y.

The EQ-5D-Y Composite and VAS scores both demonstrated responsiveness in that they improved significantly in children who were expected to show improvement, i.e. the AI and the CI children, with a medium effect. This implies that the EQ-5D-Y is a useful measure to monitor change over time and that, even in relatively small numbers of children such as the CI group, a change can be detected. Similarly a moderate effect size for EQ-5D-Y VAS, was calculated in a study comparing responsiveness of the EQ-5D-Y, KIDSCREEN-10 and KINDL- R, in a clinical sample of children with chronic conditions. However this study only found limited changes in dimension scores over time [30].

All therapists administrating the EQ-5D-Y found it easy and quick (five minute) to administer and reported that it provided them with information on the child’s HRQoL that they were previously unaware of, particularly on the less obviously observable dimensions of Pain or Discomfort and Worried, Sad or Unhappy domains.

### Limitations and recommendations

The sample size calculation of 190 children in total was based an anticipated moderate effect size between groups, however the results could have been biased by the small numbers in the two chronically ill groups.

Further qualitative research is recommended to clarify how healthy children interpreted “Mobility (walking about)”, “Usual Activities” and “Pain or Discomfort”, as there was limited reliability for the Usual Activity dimension and the EQ-5D-Y scores for Mobility and Pain or Discomfort were not associated with other outcome measures assessing the same construct.

The dimensions yielded intuitively correct results in the chronically ill children, but more research is required to understand the adaptations these children adopt in adapting to their situation and evaluating their overall HRQoL with a high VAS score similar to the healthy children.

## Conclusions

Despite the limitations in sample size, the problems the healthy children encountered in completing the EQ-5D-Y and possible adaptation occurring in chronically ill children, the study did yield useful information. The EQ-5D-Y fulfilled the psychometric requirements in the acutely ill children did not perform as well in the other groups. As it is short, responsive to change and acceptable to the users, it is recommended that it could be used as a routine measure within an acute care setting, as well as an outcome measure to monitor the impact of interventions. It should be used with caution in healthy children and children with chronic health conditions.

## Abbreviations

AI: Acutely ill group of children
CI: Chronically ill group of children with chronic health conditions
EQ-5D: European Quality of Life (EuroQol) five dimension - adult version
EuroQoL Group: The EuroQol group association comprises a network of international, multilingual, multidisciplinary researchers
FPS: Faces pain scale
HIV: Human immunodeficiency virus
HRQoL: Health related quality of life
ICC: Intraclass correlation coefficient
LAM: Looking after myself
MS: Mainstream school children
PedsQL: Paediatric quality of life inventory version 4.0 generic core scales
QALY: Quality adjusted life years
SD: Standard deviation
SS: Special school children with chronic physical disabilities
VAS: Visual analogue scale
WeeFIM: Paediatric functional independence measure

## Footnotes

1
http://yatani.jp/teaching/doku.php?id=hcistats:wilcoxonsigned.
